# Essential Mineral Content (Fe, Mg, P, Mn, K, Ca, and Na) in Five Wild Edible Species of *Lactarius* Mushrooms from Southern Spain and Northern Morocco: Reference to Daily Intake

**DOI:** 10.3390/jof8121292

**Published:** 2022-12-10

**Authors:** Alejandro R. López, Marta Barea-Sepúlveda, Gerardo F. Barbero, Marta Ferreiro-González, José Gerardo López-Castillo, Miguel Palma, Estrella Espada-Bellido

**Affiliations:** 1 Department of Analytical Chemistry, Faculty of Sciences, Agrifood Campus of International Excellence (ceiA3), IVAGRO, University of Cadiz, 11510 Puerto Real, Spain; 2 Unidad de Protección de la Salud, Distrito Sanitario Granada-Metropolitano, Consejería de Salud y Familias, Junta de Andalucía, 18150 Gójar, Spain

**Keywords:** wild edible mushrooms, *Lactarius*, essential metals, southern Spain, northern Morocco, recommended daily intake

## Abstract

Mushroom consumption has increased in recent years due to their beneficial properties to the proper functioning of the body. Within this framework, the high potential of mushrooms as a source of essential elements has been reported. Therefore, the present study aims to determine the mineral content of seven essential metals, Fe, Mg, Mn, P, K, Ca, and Na, in twenty samples of mushrooms of the genus *Lactarius* collected from various locations in southern Spain and northern Morocco, by FAAS, UV-Vis spectroscopy, and ICP-OES after acid digestion. Statistics showed that K was the macronutrient found at the highest levels in all mushrooms studied. ANOVA showed that there were statistically significant differences among the species for K, P, and Na. The multivariate study suggested that there were differences between the accumulation of the elements according to the geographic location and species. Furthermore, the intake of 300 g of fresh mushrooms of each sample covers a high percentage of the RDI, but does not meet the recommended daily intake (RDI) for any of the metals studied, except for Fe. Even considering these benefits, the consumption of mushrooms should be moderated due to the presence of toxic metals, which may pose health risks.

## 1. Introduction

Over the years, mushrooms have played a fundamental role in the recipes of many cultures, where they have been considered a delicacy due to their unique aroma, texture, and flavors [1]. Mushrooms have been considered healthy foods for human health due to their numerous beneficial properties for the organism, since they are rich in carbohydrates, proteins, vitamins, or fatty acids [2]. In addition, they contain phenolic acids and secondary metabolites that give them antibacterial, antiviral, antioxidative, anti-inflammatory, or anticarcinogenic properties [2,3,4,5]. Several studies are currently underway on the nutritional properties of mushrooms, and among other peculiarities, it has been shown that they are involved in regulating various biological processes and reducing diseases such as Alzheimer’s and cancer [6,7,8]. This has increased interest in the consumption of mushrooms in countries where they were not previously part of the diet, such as Spain, Denmark, and Sweden [9,10]. This attention has also been boosted by the population’s emerging proclivity to incorporate new foods into their diets, allowing them to adopt healthier eating habits for the proper functioning of the organism [3].

Among all the edible mushrooms, only a few are cultivated for commercialization, such as Agaricus bisporus or mushrooms of the genus Pleurotus [11]. However, the demand for wild edible mushrooms such as Boletus edulis, Macrolepiota procera, or mushrooms of the genus Lactarius has increased in the last years. This fact is due to their numerous beneficial properties and their limited availability, as they are seasonal mushrooms, to the point that their commercial value has reached, and may even exceed, the value of the timber trade [12]. This is apparent in the central and northern regions of Spain, where mushrooms are widespread in Pinus pinaster forests, especially Lactarius mushrooms. In this context, mushrooms of the genus Lactarius represent a source of economic income in these regions, as they are the most consumed and traded wild mushrooms locally and nationally [13]. All this has increased both mycological tourism and the consumption of this food.

Currently, many studies show that mushrooms are a rich source of essential elements, such as P, Mg, or Fe [4,11,14,15,16,17,18,19,20,21,22,23,24,25,26], because they can be accumulated in mushrooms’ tissues and reach higher contents than the soil in which they grow [27,28]. Essential metals are those elements that are indispensable to maintain the correct functioning of the organism due to their participation in numerous functions and biological changes in the organism. These metals can be classified into macronutrients or trace minerals [29]. The former must be incorporated into the daily diet in quantities of tens or hundreds of grams, such as Ca, P, or K, while the latter are elements that the organism needs in quantities in the order of micrograms, such as Fe or Mn. All of them are indispensable in the organism, as they form part of the active sites of different enzymes that catalyze reactions involved in important processes for life, such as the respiratory chain or the dismutation of superoxide, as well as forming part of the structures that make up cell membranes [29]. Therefore, a deficit of essential metals can put the body’s health at risk, although they can also be toxic to human health if ingested in high contents [30].

At the international level, numerous articles have been published, focusing on the study of the accumulation of metals, both essential and toxic, and their importance in human health by analyzing the results from a nutritional point of view or by studying the level of toxicity. Most of these studies have focused on the determination of essential metals such as Fe, Ca, Na, or K, as well as toxic elements as Cr, Cd, Pb, Hg, or As in mushrooms from Indian or African regions [15,16,18,20,22,26], or in mushrooms from around the world such as *Boletus edulis*, *Macrolepiota procera* or *Lactarius deliciosus* collected in regions of Poland and Turkey [4,14,21,23].

Spain has always been characterized as a mycophobic country, especially in the southern region, although in recent years tourism and consumption have increased due to the great variety of plant formations and climatic conditions, especially in Andalusia [31]. Despite the growing interest in mushrooms, the studies carried out on the determination of the metal content have been scarce in Spain and, specifically, most of them have focused on the determination of non-essential and potentially toxic metals (i.e., Pb, Hg, Cd, or As) in local mushrooms, such as *Lepista nuda* or *Coprinus comatus* from Northern regions such as Galicia or Castilla y Leon [32,33,34,35,36,37,38]. Regarding the determination of essential metals, studies have been even fewer than for toxic metals, although it is of nutritional benefit to assess their content to provide a complete and objective information about the food safety of mushrooms. For example, researchers such as Haro et al. [11] have studied the content of essential metals, such as Fe, Na, P, Cu or Ca, among others, in different species from southern Spain, such as *Macrolepiota procera, Lactarius sanguifluus*, or *Lactarius deliciosus*. However, in this work, besides *Lactarius sanguifluus* and *Lactarius deliciosus*, other species belonging to the genus *Lactarius*, such as *L. vinosus, L. semisanguifluus*, and *L. rugatus*, have been analyzed. In addition, different geographical locations in the south of Andalusia have been studied, since both mushroom species and geographical location are factors that can affect the accumulation of metals by mushrooms. This fact is evidenced by the studies carried out by other authors, where the concentrations of different essential metals, such as Fe, K, or Mg among others, vary among the different species of mushrooms of the genus *Lactarius* collected in different locations in Spain, South Africa, Poland, Serbia, and Romania [4,11,14,22,39].

Therefore, due to the interest in providing valuable information about the content of essential metals in mushrooms, the present study aims to determine the content of seven essential metals (Fe, Mg, P, Mn, K, Ca, and Na) in 19 samples of wild edible mushrooms belonging to 5 mushroom species of the genus *Lactarius* (*L. deliciosus, L. rugatus, L. vinosus, L. sanguifluus,* and *L. semisanguifluus*), which were collected from Southern Andalusia and Northern Morocco. On another side, one commercially acquired sample is also analyzed due to its availability to the consumer without any kind of nutritional information about the mineral content on this label. In addition, the contribution to the recommended daily intake (RDI) of these elements provided by the consumption of the samples is evaluated.

## 2. Materials and Methods

### 2.1. Sampling

**Figure 1:**
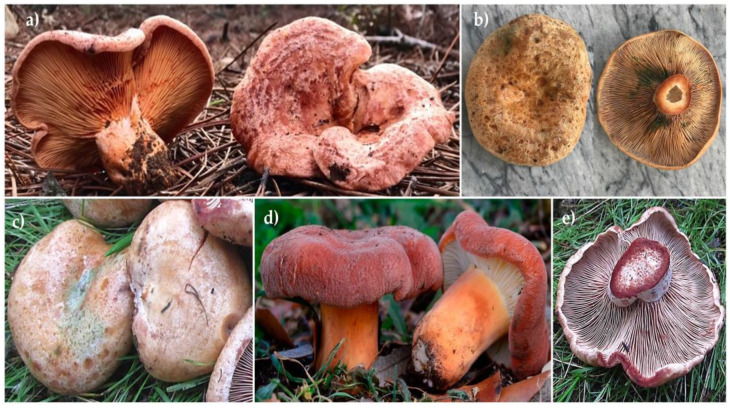
Five wild seasonal species of the genus *Lactarius* studied: (**a**) *L. deliciosus*; (**b**) *L. semisanguifluus*; (**c**) *L. sanguifluus*; (**d**) *L. rugatus*; (**e**) *L. vinosus*.

A total of twenty samples of *Lactarius* mushrooms were analyzed (Table 1). In particular, the five wild seasonal species most marketed and consumed were studied (*L deliciosus*, *L. rugatus*, *L. semisanguifluus*, *L. sanguifluus*, and *L. vinosus*) (Figure 1). The specimens were identified by experts according to their morphological characteristics that are distinctive for each species.

Nineteen of them were collected from different points in the South of Spain, located in the provinces of Cadiz, Malaga, and Granada, and locations in the North of Morocco, belonging to the cities of Tetouan and Chaouen (Figure S1 in Supplementary Data). In addition, a commercial sample of *L. deliciosus* was analyzed due to the lack of information about the mineral content on the label, specifically #11: *L. deliciosus* from Leon (Leon, Spain). To obtain a representative pool of samples, at least 10 specimens of each sample were collected to obtain a pool of samples for each area. Prior to the analysis, the collected samples were pretreated adequately. To this effect, they were washed using deionized water and then dried in an oven at 50 °C for 48 h. Finally, the dried samples were powdered using an agate mortar to homogenize them and stored in polyethylene (PE) bottles that were perfectly labeled according to the species and sampling area.

### 2.2. Chemicals and Reagents

The reagents used to carry out the acid digestions were purchased from SCP Science (Montreal, Quebec, Canada): HCl PlasmaPURE (34–37%), HNO_3_ PlasmaPURE (67–69%), and from Sigma-Aldrich (St. Louis, MO, USA): H_2_O_2_ (≥30%). On the other hand, to determine the concentration of P, the reagents were purchased from Labbox (Barcelona, Spain): H_2_SO_4_ (98%), from Panreac Química S.A (Castellar del Vallès, Barcelona, Spain): Na_2_HPO_4_ (100%), N_2_H_6_SO_4_ (99%), and from Scharlau (Sant Feliu de Llobregat, Barcelona, Spain): (NH_4_)_6_Mo_7_O_24_·4H_2_O (99%). A buffer was used to eliminate interferences associated with the measurement of magnesium, which was purchased from Merck KGaA (Darmstadt, Germany): CsCl, LaCl_3_ (100%). All the solutions were prepared using ultrapure water obtained by passing twice-distilled water through a Milli-Q system (18 MΩ/cm, Millipore, Bedford, MA, USA).

### 2.3. Digestion Procedure

The acid digestions took place employing a DigiPREP Jr block digestion system acquired from SCP Science (Montreal, Quebec, Canada). That instrument is equipped with 24 positions for 50 mL polypropylene (PE) digestion tubes (DigiTubes; SCP Science; Montreal, Quebec, Canada). The acid digestion procedure here employed has already been described in previous studies by the research group [37,38]. Firstly, the samples (0.25 g) were placed into digestion tubes. Then, 2 mL nanopure water, 2 mL HCl, and 5 mL HNO_3_ were added, and the samples were digested by applying a stepwise temperature increase procedure for 20 min up to 65 °C and maintaining this temperature for a total of 30 min. After a cooling step, 3 mL of H_2_O_2_ was added to carry out a second digestion by increasing the temperature gradually over 30 min to 110 °C, and maintaining it for a total of 60 min. To finish the procedure, the digested samples were filtered using a 0.45 μm filter and a −600 mbar vacuum port and then transferred to a 50 mL volumetric DigiTube, filling it to 50 mL with nanopure water. All samples were prepared in triplicate.

### 2.4. Elemental Analysis and Quality Control

To determine the content of Mg, Fe, and Mn in the samples of mushrooms, a flame atomic absorption spectrometer (FAAS) (Ice 3000 Series AA; Thermo Fisher Scientific; Walthman, MA, USA) was used. This instrument was equipped with a double-bundle optic system, where all lenses were covered by silica and sealed to avoid the ash entrance; an automatic alignment carousel with the capacity for 6 hollow cathode lamps; and a 50 mm universal titan burner. The instrumental parameters, as well as burner height, fuel flow, slip width, and wavelength, were optimized to determine the content of each element, and they are shown in Table 2. It is worth mentioning that in the case of Mn, to avoid interferences and a decrease in the analytical signal, it was necessary to add a releasing agent to the samples, the standards, and the certified reference material. In this case, La at a concentration equal to 1000 mg L^−1^ was employed as releasing agent.

For its part, the determination of Ca, Na and K was performed by using an Inductively Coupled Plasma Optical Emission Spectrometer (ICP-OES) (Spectrogreen FMX46; SPECTRO Analytical Instruments; Kleve, Dusseldorf, Germany). The ICP-OES used was an automatic optical emission spectrometer that provides simultaneous measurements using inductively coupled plasma excitation (argon ions) and a semiconductor-based detector system for the analysis of liquids. It offers a wide range of spectral analysis from 165 to 770 nm.

P, on the other hand, is not usually determined by FAAS or ICP-OES, as its main atomic lines, both in absorption and emission, are between 167 nm and 179 nm, a range inaccessible to conventional absorption or atomic emission spectroscopic techniques [40]. Therefore, the quantification of this element was carried out employing UV-Vis molecular absorption spectrophotometry using an adaptation of the official method recommended by the Association of Official Agricultural Chemists (AOAC) for determining phosphorus in cheese and processed cheese products. This methodology has been successfully adapted by other authors for the determination of phosphorus content in walnuts after optimization of the method for these types of samples [41]. As the concentration of hydrazine sulphate required to reduce ammonium phosphomolybdate to molybdenum blue depends on the content of P present in the samples, it is necessary to optimize the method for its adaptation to the determination of P in mushrooms. Therefore, a preliminary study was carried out to determine the optimum concentration of hydrazine sulphate with which maximum absorbance values are obtained. To carry out the experiments, 1 mL of one of the digested mushroom samples—specifically #10: *L. deliciosus* from Fuente del Espino (Granada, Spain)—was taken in a 100 mL Erlenmeyer flask. Subsequently, about 10 mL of Milli-Q water and 25 mL of one of the reagents ammonium molybdate and hydrazine sulphate were added. The mixture was heated at 140 °C for 4.5 min and allowed to cool to room temperature. After cooling, the contents were poured into a 50 mL volumetric flask and made up to the mark with ultrapure water. In addition, the content of P was determined using a double-bundle UV-Vis molecular absorption spectrophotometer (UV-Vis MAS) (V-630; Jasco; Ishikawa-Machi, Hachioji-shi, Tokyo, Japan) at 820 nm. Specifically, the optimization of the P determination method by UV-Vis MAS through the formation of the molybdenum blue complex was carried out by means of a univariate design of the hydrazine sulphate concentration, using the values of absorbance and coefficient of variation (C.V.) as selection criteria. The concentrations tested were 0.15% p/V, 0.5% p/V, 1% p/V, 2% p/V and 3% p/V, based on the concentration used by the AOAC in the determination of P in cheese [42]. The measurements were performed in triplicate following the experimental procedure explained above. The maximum absorbance value was obtained employing 1% p/V hydrazine sulphate with a low coefficient of variation (0.18%). Consequently, this concentration was selected for further experiments. Therefore, the official method for the determination of P in cheese and processed cheese products, recommended by the AOAC, was successfully adapted for application to mushroom samples, after optimization of the concentration of hydrazine sulfate.

Before the analyses, it was necessary to verify the analytical methodology employed for Na, K, Ca, Fe, Mg, P, and Mn. For this purpose, a certified reference material (CRM), specifically Tea leaves INCTL-TL-1 (Institute of Nuclear Technology and Chemistry; Warsaw, Poland), was used. The recovery levels found in the reference material (INCT-TL-1) were in an acceptable range of 92–108% for the studied elements, and precision (0.76–2.79%) obtained was satisfactory. On the other hand, the limits of detection (LOD) and the limits of quantification (LOQ) were ascertained at the levels of 0.003–0.046 mg·L^−1^ and 0.010–0.154 mg·L^−1^, respectively, for all the studied elements.

### 2.5. Recommended Daily Intake of Metals

Macronutrients take a fundamental role in assuring the correct functioning of the body. However, a certain amount of each element is required to achieve that, known as recommended daily intake. Levels above or below the recommended daily intake can lead to different health-related disorders, as well as increase the risk of various diseases. Therefore, to determine whether the consumption of mushrooms of the genus *Lactarius* collected both in southern Spain and northern Morocco satisfies the recommended daily intake of the different metals evaluated in this study, the mineral content determined in mushroom samples was compared with the recommended daily intake values for each element: Fe, Mn, P, Mg, Ca, P, and Na.

The estimation of the daily intake of each element (M), provided by the mushrooms, was calculated according to the following expression:

M = C × I(1)

where C is the metal content present in mushroom samples, expressed in mg Kg^−1^, and I is the amount of dry matter provided by the intake of mushrooms, measured in Kg dry matter per day, assuming a tolerable daily intake of 300 g of fresh mushrooms, which contains 30 g of dry matter [16].

The contribution of all mushroom samples analyzed to the recommended daily intake was calculated as the percentage between the estimation of the daily intake of each metal provided by mushroom and the RDI value according to the Spanish Federation of Nutrition, Food and Dietetic Societies (FESNAD) [43], following the expression:

A = (M/RDI) × 100(2)

where M is the estimation of the daily intake of each element provided by the mushrooms, expressed in mg day^−1^, and RDI is the recommended daily intake, expressed in mg day^−1^.

### 2.6. Data Analysis and Software

To extract the raw data from the instrumental equipment, the software used were: ICP Analyzer-Pro software (Kleve, Dusseldorf, Germany) for ICP-OES, Solaar (Waltham, MA, USA) for FAAS, and Spectra Manager (Ishikawa.Machi, Hachioji.shi, Tokyo, Japan) for UV-Vis MAS. To carry out the univariant statistical study, STATGRAPHICS Centurion 18 version 18.1.16 (StatPoint Technologies Inc., Warrenton, VA, USA) was employed. On the other hand, to perform the multivariate analysis of the data, RStudio software (R version 4.1.2, Boston, MA, USA) was employed. Here, a hierarchical cluster analysis (HCA), an unsupervised tool for pattern recognition in the dataset, was performed using the *hclust* function of the *stats* package (version 4.1.2). Linkage method selection for the HCA was established by computing the agglomerative coefficient of different linkage methods (Average, Single, Complete, and Ward) using the *agnes* function of the *cluster* package (version 3.1.3). The HCA results were represented using the *factoextra* package (version 1.0.7). The rest of the graphs presented in this work were made with Microsoft Office software (Redmond, WA, USA).

## 3. Results and Discussion

### 3.1. Essential Mineral Content

The content of essential metals in each sample of mushroom is shown in Table 3. Mn was the only element that was below the limit of detection (LOD) in the seven mushroom samples. In general terms and according to the results obtained, K is the element found in the highest levels in the samples, followed by P, with Mn found in the lowest levels. On the one hand, K is the main cation in the body and is involved in many functions such as nerve impulse and muscle contraction. K deficiency leads to muscle weakness, cardiac arrhythmias, kidney stones, risk of heart attacks, etc. [44]. On the other hand, P is one of the major components of bones and teeth, regulates pH, buffers acid or base excess, and is a major component of biological membranes. It is very difficult for P deficiency to occur, since this element is present in most of the foods that make up the human diet. However, alcohol or too rapid a breakdown of fat can lead to hypophosphatemia, characterized by anorexia, anemia, muscle weakness, bone pain, or rickets [44]. For its part, Mn acts as a cofactor in numerous metalloenzymes involved in reproduction, metabolism, and bone growth [30]. Mn is also essential for regulating blood sugar, regulating immune function, and promoting blood clotting [45]. Mn deficiency can cause hypocholesterolemia and scaly dermatitis [44].

Potassium: According to the results, the K content in the samples ranged from 13,845 mg Kg^−1^ to 45,635 mg Kg^−1^ with an average of 25,398 mg Kg^−1^. The highest level of K was measured in sample #20: *L. rugatus* (45,635 ± 672 mg Kg^−1^) from Cortes de la Frontera (Malaga, Spain). However, the lowest content belongs to sample #2: *L. deliciosus* (13,845 ± 1.00 mg Kg^−1^) from Talassemtane (Chaouen, Morocco). The K levels determined by other authors were in the range 16,290–74,000 mg Kg−^−1^ in *L. deliciosus* [11,14,22] and the contents were around 15,100 mg Kg^−1^ in *L. sanguifluus* [11]. Therefore, the K content found in other papers agrees with the results of this study.

Magnesium: In the case of the determination of Mg, the mean value of the content in the samples was 1034 mg Kg^−1^, with a minimum value of 775 mg Kg^−1^ and a maximum value of 1575 mg Kg^−1^. The mushrooms with the highest level of Mg belong to sample #18: *L. sanguifluus* (1575 ± 60.4 mg Kg^−1^) from Sierra de Huetor (Granada, Spain). However, sample #19: *L. rugatus* from Cortes de la Frontera (Malaga, Spain) had the lowest content of Mg (775 ± 22.3 mg Kg^−1^). The Mg content for *L. sanguifluus* found in the literature was around 1100 mg Kg^−1^ [11], while for *L. deliciosus* it ranged from 800–1190 mg Kg^−1^ [11,14,22]. Those results agree with the Mg levels determined in the present study.

Calcium: Regarding the Ca, the mean value of the content in the samples is 463.32 mg Kg^−1^. The highest level was determined in sample #2: *L. deliciosus* (995 ± 0.06 mg Kg^−1^) from Talassemtane (Chaouen, Morocco), and the lowest one belongs to sample #19: *L. rugatus* (123 ± 8.78 mg Kg^−1^) from Cortes de la Frontera (Malaga, Spain). The Ca content levels reported by other authors were in the range of 78.4–460 mg Kg^−1^ in *L. deliciosus* [11,14,22,46] and around 74.7 mg Kg^−1^ in *L. sanguifluus* [11]. It can be observed that levels of Ca determined in this study are higher than the content reported for *L. sanguifluus* and some samples of *L. deliciosus* in the literature.

Sodium: On the other hand, the highest level of Na was found in sample #13: *L. vinosus* (1008 ± 0.04 mg Kg^−1^) from Pinar el Colorado (Cadiz, Spain) while the lowest content was found in sample #11: *L. deliciosus* (15.9 ± 0.004 mg Kg^−1^) from Leon (Leon, Spain), (i.e., commercial sample). The mean value of the Na levels was 208 mg Kg^−1^. In the literature, it was found that Na content for *L. deliciosus* was in the range of 30.3–430 mg Kg^−1^ [11,14,22,46] and for *L. sanguifluus* was around 74.4 mg Kg^−1^ [11]. Those results agree with the range of levels determined in this study.

Iron: Given the results, the values of Fe content in the mushroom samples range from 56.5 mg Kg^−1^ to 1245 mg Kg^−1^, with a mean value of 335 mg·Kg^−1^. The highest Fe content was found in sample #10: *L. deliciosus* (1245 ± 2.49 mg Kg^−1^) from Fuente del Espino (Granada, Spain). However, the lowest Fe content belongs to sample #6: *L. deliciosus* (56.5 ± 0.11 mg Kg^−1^) from Sendero el Palancar (Cadiz, Spain). Other authors have reported that the levels of iron for *L. deliciosus* were in the range of 40–1190 mg Kg^−1^ [4,11,14,22] and for *L. sanguifluus* the level was around 602 mg Kg^−1^ [11]. The results obtained in this study are in accordance with the literature.

Manganese: The range of Mn content present in the samples is between a minimum and a maximum value of 6.68 mg Kg^−1^ and 43.4 mg Kg^−1^, respectively, with an average of 25.5 mg Kg^−1^. The highest level of Mn belongs to sample #17: *L. semisanguifluus* (43.4 ± 4.38 mg Kg^−1^) from Sierra de Huetor (Granada, Spain). Meanwhile, the sample with the lowest Mn content is sample #11: *L. deliciosus* (6.68 ± 0.47 mg Kg^−1^) from Leon (Leon, Spain), (i.e., commercial sample). The range of Mn content found in the literature was 3.85–41.0 mg Kg^−1^ for *L. deliciosus* [4,14,22]. It can be seen that our results agree with those obtained by other authors.

Phosphorus: Finally, the P levels in the samples of mushrooms of the genus *Lactarius* range from 1918 mg Kg^−1^ to 5962 mg Kg^−1^, with a mean value of 3909 mg Kg^−1^. The mushrooms with the highest content belong to sample #18: *L. sanguifluus* (5962 ± 186 mg Kg^−1^) from Sierra de Huetor (Granada, Spain). However, the lowest level was found in sample #20: *L. rugatus* (1918 ± 127 mg Kg^−1^) from Cortes de la Frontera (Malaga, Spain). Haro et al. reported that the P level in *L. deliciosus* was around 6270 mg Kg^−1^, while the content in *L. sanguifluus* was around 5070 mg Kg^−1^ [11]. Those values are similar to our results. The high P content found in the mushroom samples analyzed is noteworthy. For this reason, a comparison with the “Nutrient Guide” food composition table was made [47], which compiles the nutritional values of different foods frequently found in the Mediterranean diet according to data from different departments, ministries, and organizations. Based on this information, other mushrooms, belonging to other genera and species different from those analyzed in this study, such as *Shiitake* mushrooms or *Chanterellas*, present P levels of 2940 mg Kg^−1^ and 570 mg Kg^−1^, respectively, which are below the range determined for mushrooms of the genus *Lactarius*. Another interesting piece of information in the Nutrient Guide is the P content present in different types of nuts, which are characterized by their high P level. These foods have a P content of between 380 mg Kg^−1^ and 5930 mg Kg^−1^, like the mineral content of the mushroom samples studied.

In accordance with all the results previously mentioned, it is worth noting that sample #18: *L. sanguifluus* from Sierra de Huetor (Granada, Spain) presents the highest level of the majority elements studied.

### 3.2. Statistics and Multivariate Analysis

First, to establish whether there are statistically significant differences between species in terms of the mineral content, a univariate statistical study based on analysis of variance (ANOVA, 95% significant level) was conducted. A post hoc test, specifically the least significant difference test, was performed when statistically significant differences were obtained. The results obtained through the ANOVA test (Table S1. Supplementary Data) showed that nonsignificant differences were found for Fe (*F* = 0.28 and *p*-value = 0.887), Mg (*F* = 2.17 and *p*-value = 0.122), Mn (*F* = 2.52 and *p*-value = 0.085) and Ca (*F* = 1.09 and *p*-value = 0.398) while significant differences were found for K (*F* = 4.01 and *p*-value < 0.05) specifically between *L. deliciosus* and *L. rugatus*, *L. rugatus* and *L. semisanguifluus*, and *L. rugatus* and *L. vinosus*; for P (*F* = 3.72 and *p*-value < 0.05) between *L. deliciosus* and *L. rugatus*, *L. rugatus* and *L. sanguifluus*, and *L. rugatus* and *L. vinosus*; and for Na (*F* = 7.72 and *p*-value < 0.05) between the species *L. deliciosus* and *L. vinosus*, *L. rugatus* and *L. vinosus*, *L. sanguifluus* and *L. vinosus*, and *L. semisanguifluus* and *L. vinosus*.

**Figure 2:**
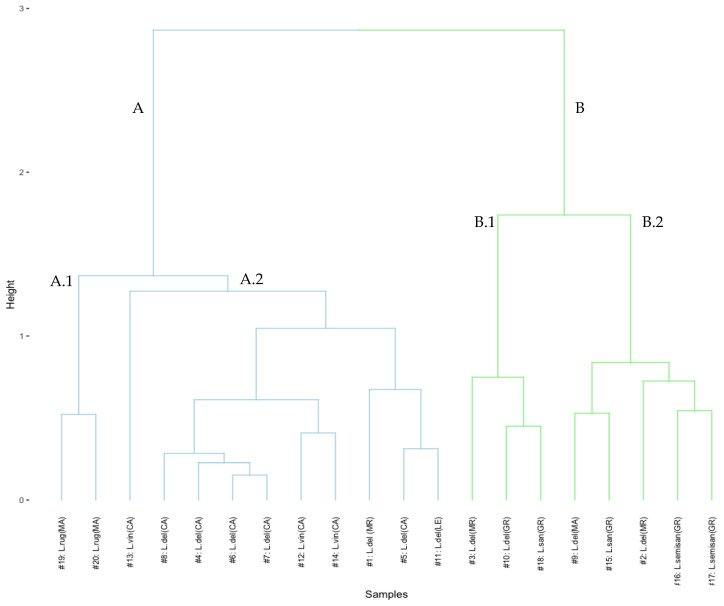
Graphical display of the resulting HCA dendrogram using Euclidean distance and Ward’s method to identify clustering trend patterns among the studied mushroom species based on the content of the seven macronutrients determined.

The clustering trend of mushroom samples according to their mineral content was evaluated. For this purpose, a multivariate analysis technique, HCA, was used to establish recognition patterns in the dataset that would allow us to observe groupings by species, geographic location, or both. The data matrix D*_mxn_*, where *m* is the number of macronutrients studied (*m* = 7) and *n* is the number of mushrooms analyzed (*n* = 20), was normalized using min-max normalization. For this HCA, Euclidean distance was selected as the distance measure, and Ward’s method as the linking method. The choice of the linkage method was determined from the comparison of the agglomerative coefficient of the different methods (Average, Full, Single, and Ward). Agglomerative coefficients close to 1 would indicate a stronger clustering structure. In this case, Ward’s method presented the highest agglomerative coefficient (0.83) among the linkage methods evaluated. The results obtained using HCA are represented in the dendrogram shown in Figure 2. According to the results, it can be observed that the samples tend to cluster into two main clusters: A and B. On the one hand, cluster A is divided into two subclusters. The subcluster A.1 contains the two samples of *L. rugatus* collected in the province of Malaga, Spain. Meanwhile, subcluster A.2 is constituted by all the samples of *L. deliciosus* and *L. vinosus* collected in the province of Cadiz, Spain. At the same time, a sample of *L. deliciosus* collected in Morocco and the commercially acquired sample of *L. deliciosus* are grouped in this cluster. It is noteworthy to mention that the samples of *L. vinosus* are separated from those of *L. deliciosus* within this subcluster. On the other hand, cluster B is also divided into two subclusters. It is noteworthy that all the samples collected in Granada (Spain) are grouped in this cluster. However, the tendency of grouping according to species and/or geographic location is less pronounced than in cluster A. In this sense, subcluster B.1 is composed of a sample of *L. sanguifluus* collected in the province of Granada (Spain) and two samples of *L. deliciosus*, one from Morocco and the other from the province of Granada (Spain). Subcluster B.2 consists of samples of *L. sanguifluus*, *L. deliciosus*, and *L. semisanguifluus*. However, it is possible to observe how the two samples of *L. semisanguifluus* are separated from the rest within the same subcluster. Thus, it is possible to observe that although environmental factors influence the bioavailability of macronutrients in the medium, intrinsic factors such as species have a greater influence on the phenomenon of macronutrient accumulation in mushrooms. However, this grouping is not completely consistent since there is no perfect clustering.

### 3.3. Recommended Daily Intake

The reference values of the RDIs for the different elements were obtained by the Spanish Federation of Nutrition, Food and Dietetic Societies (FESNAD). For calculations, the RDI of children aged 10 to 13 years—as it is assumed that younger children do not consume a daily intake of 300 g of fresh mushrooms—and adults aged 20 to 59 years were employed.

After calculating the content of essential metals incorporated into the body by consuming a daily intake of 300 g of fresh mushroom of each sample analyzed, it can be seen that none of them on their own satisfies the recommended daily intake for the metals determined, except for Fe (Table 4). For this last metal, samples #2: *L. deliciosus* from Talassemtane (Morocco) and #16: *L. semisanguifluus* from Puerto de la Mora (Granada, Spain) exceed the RDI values for men; samples #3: *L. deliciosus* from Dchar Akjiouene (Chaouen, Morocco), #10: * L. deliciosus* from Fuente del Espino (Granada, Spain), and #18: *L.sanguifluus* from Sierra de Huetor (Granada, Spain) exceed the RDI values for men, women, and children; and samples #13: *L. vinosus* from Pinar el Colorado (Cadiz, Spain) and #17: *L. semisanguifluus* from Sierra de Huetor (Granada, Spain) exceed the RDI for men and children according to the FESNAD. Fe is the most abundant trace element in the body, as it is present in numerous proteins, such as hemoglobin, as well as in various enzymes or cytochromes involved in redox reactions [30]. Due to its numerous oxidation states, and its broad capacity to form coordination compounds, iron is present throughout the biological system, performing different functions [44]. An Fe deficiency in the human body can cause health problems, the most common being anemia. On the other hand, an excess of Fe can lead to acute toxicity, accompanied by gastrointestinal disorders, such as diarrhea or vomiting, or affect the proper functioning of the liver, the central nervous system, or the cardiovascular system [44]. Therefore, considering that some of the analyzed mushroom samples exceed the RDI levels for Fe, the excessive and prolonged consumption of mushrooms of the species and geographical locations indicated could pose a health risk in terms of this element.

On the other hand, it is observed that the amount present in the mushroom samples for the different elements studied covers a high percentage of the RDI of these elements, except for Ca and Na, as shown in Table 5. Therefore, it should be noted that for all metals except Fe, only the daily intake of 300 g of fresh mushrooms would be insufficient to satisfy the RDI, so to achieve this, it would be necessary to supplement the diet with foods rich in these elements.

### 3.4. Health Risk Due to Toxic Metal Content

From the point of view of nutritional interest, the study of the content of essential elements in mushrooms is indispensable. However, the concentration of toxic metals is another factor to be taken into account. Therefore, although the mushrooms studied have high levels of macronutrients and trace elements, it is of vital importance to know the contribution of toxic metals by their intake in order to determine whether the consumption may pose a health risk.

**Figure 3:**
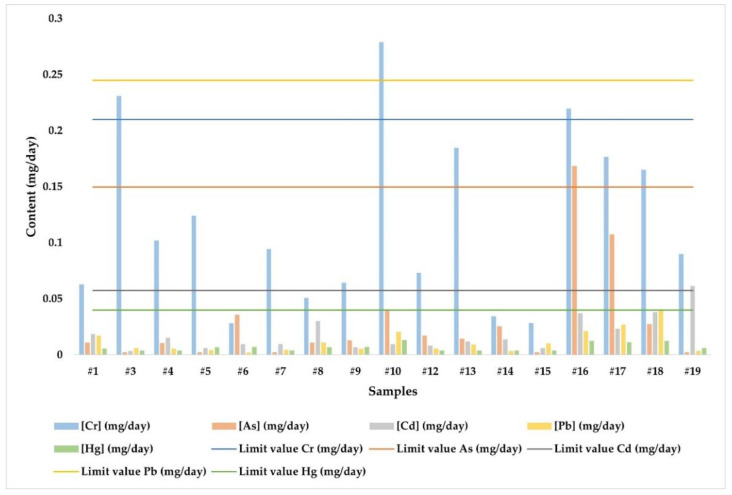
Representation of the toxic metal content of mushrooms of the genus *Lactarius* and the recommended daily limits.

Previous studies carried out by our research group [37] regarding the levels of As, Cd, Cr, Pb, and Hg in the same samples of mushrooms evaluated in this study and in the same geographical locations have allowed us to carry out a comparison between the contents of macronutrients and potentially toxic metals. As previously mentioned in this research, it was observed that sample #10: *L. deliciosus* from Fuente del Espino (Granada, Spain) was one of the mushrooms with the highest contents of the metals studied. However, as shown in the previous study in regards to toxic elements [37], this sample may pose a risk to human health because it exceeds the recommended daily doses of Cr, whose hexavalent state, (i.e., Cr(VI)), is classified as carcinogenic due to its mutagenic properties [48]. The same occurs with sample #3: *L. deliciosus* from Dchar Akjiouene (Chaouen, Morocco), which exceeds the daily limits established for Cr; sample #16: *L. semisanguifluus* from Puerto de la Mora (Granada, Spain), which exceeds the daily limits established for As and Cr; and sample #19: *L. rugatus* from Cortes de la Frontera (Malaga, Spain), which exceeds the daily limits established for Cd. All this information has been displayed graphically in Figure 3, which shows the content of toxic metals in the samples studied and the recommended daily limits, with the exception of samples #2: *L. deliciosus* from Talassemtane (Morocco), commercial sample #11: *L. deliciosus* from Leon (Leon, Spain), and #20: *L. rugatus* from Cortes de la Frontera (Malaga, Spain), which were not previously analyzed in the mentioned work, and therefore, it would be necessary to determine the Cr, As, Pb, Hg, and Cd content in order to ensure safe consumption in terms of toxic metals.

Therefore, along with the preliminary conclusions presented here, the need for continuous monitoring taking into account spatial, temporal, and seasonal variations should be emphasized as a future effort.

As it is well recognized, mushrooms are becoming an increasingly popular food in the diet, and especially in vegetarian and vegan trends. From the standpoint of their macronutrient content, the study presented here suggests that to achieve the necessary daily levels of K, Na, Ca, Fe, P, Mg, and Mn, wild edible mushrooms should be consumed together with other foods during the mushrooming season. At the same time, the comparison presented herein with the levels of toxic metals found in mushrooms suggests that their consumption should be moderate. Thus, both studies on macronutrient content and potentially toxic elements are necessary to provide valuable information on how mushrooms should be consumed safely and wisely.

## 4. Conclusions

This study reveals that the intake of these mushroom samples largely satisfies the RDI of the different elements determined, except for Ca and Na. Some samples exceeded the limit value for Fe, which may pose a risk to human health, as an excess of essential metals can also affect the proper functioning of the body and lead to serious adverse effects. In relation to the statistical analysis of the data, on the one hand, the one-way ANOVA test indicated that there were no statistically significant differences between the different species in terms of mineral content for Fe, Mn, and Ca, while there were significant differences for K, P and Na. On the other hand, the results obtained with HCA suggest that although clustering is not perfect, samples tend to cluster by species with a stronger influence than geographic location.

## Figures and Tables

**Table 1:** List of all samples of *Lactarius* mushrooms analyzed with their corresponding sample ID, specie, specimen number (*n*), collection location, sampling year, and geographical coordinates.

Sample ID	Specie	Location/Year of Collection	Latitude	Longitude
#1	*L. deliciosus* (*n* = 20)	Alimadene (Tetouan, Morocco)/2017	35° 16′ 8.2′′ N	5° 26′ 7.4′′ W
#2	*L. deliciosus* (*n* = 26)	Talassemtane (Chaouen, Morocco)/2017	35° 04′ 36.6′′ N	5° 13′ 49.2′′ W
#3	*L. deliciosus* (*n* = 26)	Dchar Akjiouene (Chaouen, Morocco)/2017	35° 04′ 36.9′′ N	5° 13′ 49.5′′ W
#4	*L. deliciosus* (*n* = 16)	Dehesa de las Yeguas (Cadiz, Spain)/2017	36° 33′ 7.0′′ N	6° 07′ 50.9′′ W
#5	*L. deliciosus* (*n* = 18)	Pinar del Rey (Cadiz, Spain)/2017	36° 14′ 6.3′′ N	5° 23′ 55.9′′ W
#6	*L. deliciosus* (*n* = 19)	Sendero El Palancar (Cadiz, Spain)/2017	36° 14′ 50.5′′ N	5° 33′ 39.9′′ W
#7	*L. deliciosus* (*n* = 14)	Jimena de la Frontera (Cadiz, Spain)/2017	36° 26′ 23.2′′ N	5° 27′ 31.0′′ W
#8	*L. deliciosus* (*n* = 12)	Puerto Real (Cadiz, Spain)/2018	36° 31′ 21.7′′ N	6° 07′ 40.7′′ W
#9	*L. deliciosus* (*n* = 20)	Cortes de la Frontera (Malaga, Spain)/2018	36° 34′ 13.3′′ N	5° 24′ 3.7′′ W
#10	*L. deliciosus* (*n* = 22)	Fuente del Espino (Granada, Spain)/2018	37° 04′ 9.4′′ N	3° 05′ 26.6′′ W
#11	*L. deliciosus* (*n* = 15)	Leon (Leon, Spain)/2018	-	-
#12	*L. vinosus* (*n* = 11)	Dehesa de las Yeguas (Cadiz, Spain)/2017	36° 33′ 22.4′′ N	6° 07′ 40.7′′ W
#13	*L. vinosus* (*n* = 13)	Pinar El Colorado (Cadiz, Spain)/2017	36° 20′ 13.9′′ N	6° 05′ 45.9′′ W
#14	*L. vinosus* (*n* = 12)	Puerto Real (Cadiz, Spain)/2018	36° 31′ 21.7′′ N	6° 07′ 40.7′′ W
#15	*L. sanguifluus* (*n* = 16)	Cortes de la Frontera (Malaga, Spain)/2018	36° 34′ 12.5′′ N	5° 24′ 4.4′′ W
#16	*L. semisanguifluus* (*n* = 16)	Puerto de la Mora (Granada, Spain)/2018	37° 16′ 58.9′′ N	3° 27′ 36.6′′ W
#17	*L. semisanguifluus* (*n* = 19)	Sierra de Huetor (Granada, Spain)/2018	37° 16′ 45.6′′ N	3° 26′ 21.9′′ W
#18	*L. sanguifluus* (*n* = 21)	Sierra de Huetor (Granada, Spain)/2018	37° 17′ 19.2′′ N	3° 27′ 24.9′′ W
#19	*L. rugatus* (*n* = 12)	Cortes de la Frontera (Malaga, Spain)/2018	36° 33′ 58.0′′ N	5° 24′ 28.5′′ W
#20	*L. rugatus* (*n* = 12)	Cortes de la Frontera (Malaga, Spain)/2018	36° 35′ 2.8′′ N	5° 23′ 37.3′′ W

**Table 2:** Optimized FAAS instrumental parameters to determine Mg, Fe, and Mn.

FAAS Parameters	Metal		
	Mg	Fe	Mn
Burner height (mm)	7.8	11.8	8.6
Fuel flow (L min^−1^)	0.9	1.0	0.9
Slit width (nm)	0.5	0.2	0.2
Wavelength (nm)	285.2	248.3	279.5

**Table 3:** Mineral content in samples of *Lactarius* mushrooms analyzed (mg Kg^−1^ dry weight). Values are expressed as mean ± standard deviation (*n* = 3).

ID	Fe	Mg	Mn	P	K	Ca	Na
#1	224 ± 1.79	1093 ± 1.09	13.8 ± 0.40	4822 ± 190	-	-	-
#2	401 ± 0.80	1024 ± 3.07	27.6 ± 0.44	4475 ± 17.5	13,845 ± 1.00	995 ± 0.06	119 ± 0.01
#3	1132 ± 1.13	1146 ± 5.73	42.9 ± 0.26	5085 ± 60.4	20,617 ± 1.00	661 ± 0.03	64.6 ± 0.01
#4	114 ± 0.57	899 ± 2.67	<LOD	3179 ± 88.1	21,771 ± 1.00	248 ± 0.01	87.9 ± 0.01
#5	129 ± 0.90	915 ± 4.58	10.6 ± 0.10	4442 ± 359	21,922 ± 1.00	279 ± 0.01	178 ± 0.01
#6	56.5 ± 0.11	827 ± 2.48	<LOD	3387 ± 115	20,980 ± 1.00	253 ± 0.01	213 ± 0.01
#7	103 ± 0.31	796 ± 1.59	<LOD	2957 ± 39.1	19,495 ± 1.00	299 ± 0.01	290 ± 0.04
#8	84.0 ± 1.26	942 ± 4.71	<LOD	3334 ± 43.1	26,505 ± 1.00	400 ± 0.01	239 ± 0.01
#9	240 ± 1.68	1107 ± 2.21	28.3 ± 0.23	4630 ± 66.4	24,910 ± 1.00	339 ± 0.01	61.8 ± 0.10
#10	1245 ± 2.49	1433 ± 7.16	32.1 ± 0.10	5831 ± 323	31,744 ± 1.00	558 ± 0.02	28.0 ± 0.003
#11	84.0 ± 1.26	1081 ± 7.57	6.68 ± 0.47	4368 ± 105	22,519 ± 1.00	144 ± 0.01	15.9 ± 0.004
#12	168 ± 0.34	805 ± 1.61	<LOD	3868 ± 93.1	21,921 ± 4.00	318 ± 0.06	505 ± 0.10
#13	413 ± 1.65	886 ± 1.77	<LOD	4032 ± 223	23,014 ± 1.00	723 ± 0.02	1008 ± 0.04
#14	79.7 ± 10.3	1071 ± 15.9	<LOD	4373 ± 88.4	26,635 ± 28.3	463 ± 6.89	444 ± 1.33
#15	77.4 ± 6.36	1042 ± 2.90	38.2 ± 2.37	3734 ± 44.7	26,196 ± 849	330 ± 10.59	455 ± 16.31
#16	323 ± 10.8	1247 ± 26.7	25.5 ± 1.43	2428 ± 10.4	26,744 ± 743	591 ± 14.94	39.2 ± 4.44
#17	412 ± 2.03	1138 ± 4.70	43.4 ± 4.38	3605 ± 47.6	22,233 ± 50.5	672 ± 22.69	37.2 ± 1.58
#18	900 ± 58.2	1575 ± 60.4	23.1 ± 1.78	5962 ± 186	30,101 ± 1092	758 ± 0.98	39.3 ± 0.70
#19	79.9 ± 8.76	775 ± 22.3	6.76 ± 0.01	2200 ± 82.2	36,890 ± 354	123 ± 8.78	108 ± 4.48
#20	190 ± 8.66	942 ± 45.7	24.1 ± 0.58	1918 ± 127	45635 ± 672	246 ± 8.05	54.2 ± 0.10

**Table 4:** Daily intake of metals in samples of *Lactarius* mushrooms analyzed and recommended daily intake.

Metal	Daily Intake of Metals (mg/day)										Recommended Daily Intake (mg/day)		
	#1	#2	#3	#4	#5	#6	#7	#8	#9	#10	Children	Adults	
												Men	Women
**Fe**	6.71	12.0	34.0	3.42	3.86	1.70	3.09	2.52	7.20	37.4	12	9.5	18
**Mg**	32.8	30.7	34.4	26.7	27.5	24.8	23.9	28.2	33.2	43.0	280	350	300
**Mn**	0.413	0.829	1.29	n.d.	0.318	n.d.	n.d.	n.d.	0.848	0.962	1.9	2.3	1.8
**P**	145	134	153	95.4	133	102	88.7	100	139	175	900	700	700
**K**	n.d.	415	619	653	658	629	585	795	747	952	3100	3100	3100
**Ca**	n.d.	29.9	19.8	7.43	8.37	7.58	8.98	12.0	4.31	16.7	1100	900	900
**Na**	n.d.	3.57	1.94	2.64	5.35	6.41	8.69	7.17	1.85	0.84	1500	1500	1500
**Metal**	**Daily Intake of Metals (mg/day)**										**Recommended Daily** **Intake (mg/day)**		
	**#11**	**#12**	**#13**	**#14**	**#15**	**#16**	**#17**	**#18**	**#19**	**#20**	**Children**	**Adults**	
												**Men**	**Women**
**Fe**	2.52	5.04	12.4	2.39	2.32	9.69	12.4	27.0	2.40	5.70	12	9.5	18
**Mg**	32.4	24.2	26.6	32.1	31.2	37.4	34.1	47.2	23.2	28.3	280	350	300
**Mn**	0.200	n.d.	n.d.	n.d.	1.15	0.764	1.30	0.692	0.203	0.723	1.9	2.3	1.8
**P**	131	116	121	131	112	72.8	108	179	66.0	57.5	900	700	700
**K**	676	658	690	799	786	802	667	903	1107	1369	3100	3100	3100
**Ca**	12.0	9.55	21.7	13.9	9.90	17.7	20.2	22.7	3.69	7.39	1100	900	900
**Na**	0.48	15.1	30.2	13.3	13.6	1.18	1.12	1.18	3.24	1.63	1500	1500	1500

**Table 5:** Percentage contribution interval of RDI of each metal.

Metal	Percentage Contribution Interval of RDI (%)		
	Children	Men	Women
Fe	14–311	9–208	18–393
Mg	8–17	8–16	7–13
Mn	1–68	1–72	0.9–57
P	6–20	8–26	8–26
K	13–44	13–44	13–44
Ca	0.3–3	0.4–3	0.4–3
Na	0.032–2	0.032–2	0.032–2

## Data Availability

The data presented in this study are contained within the article.

## Supplementary Materials

The following supporting information can be downloaded at: https://www.mdpi.com/article/10.3390/jof8121292/s1, Figure S1: Map with the locations of the different sampling places of the studied mushrooms; Table S1: Results of one-way ANOVA test performed with a confidence level of 95% for quantified each essential metal in the *Lactarius* mushrooms collected in northern Morocco and southern Spain.
