# Supplementary material for: Essential Mineral Content (Fe, Mg, P, Mn, K, Ca, and Na) in Five Wild Edible Species of Lactarius Mushrooms from Southern Spain and Northern Morocco: Reference to Daily Intake
Source: J Fungi (Basel). 2022 Dec 10;8(12):1292. doi: 10.3390/jof8121292 (PMC9781426; doi:10.3390/jof8121292)
Supplement: Supplementary file 1 [file jof-08-01292-s001.zip › jof-2055885-supplementary.pdf]

Essential Mineral Content (Fe, Mg, P, Mn, K, Ca, and Na) in Five Wild Edible Species of *Lactarius* Mushrooms from Southern Spain and Northern Morocco: Reference to Daily Intake

Alejandro R. López [1,a], Marta Barea-Sepúlveda [1,a], Gerardo F. Barbero [1,*], Marta Ferreiro-González [1], José Gerardo López-Castillo [2], Miguel Palma [1] and Estrella Espada-Bellido [1,*]

Supplementary Material

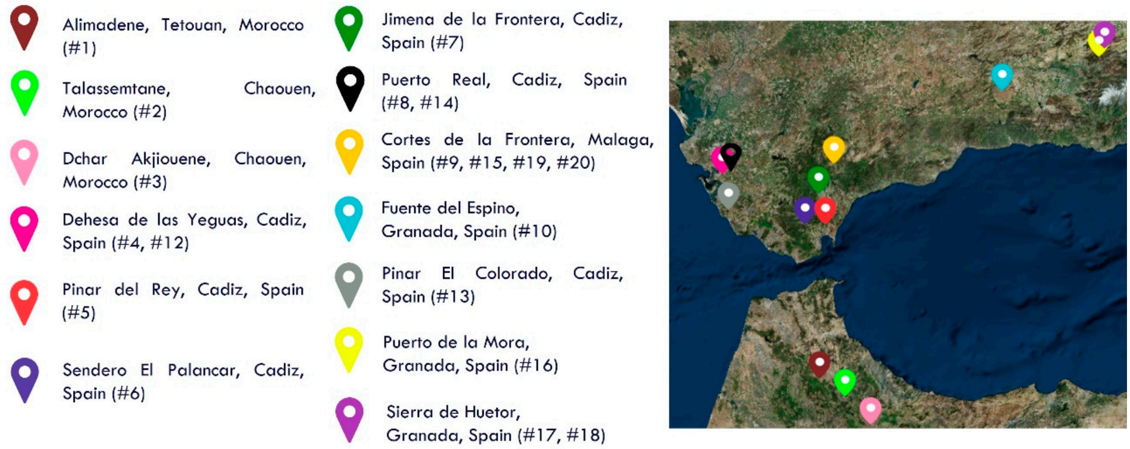

Figure S1. Map with the locations of the different sampling places of the studied mushrooms.

Table S1. Results of One-Way ANOVA test performed with a confidence level of 95% for quantified each essential metal in the *Lactarius* mushrooms collected in northern Morocco and southern Spain.

Essential metal	One-Way ANOVA
**Fe**	*F* = 0.28<br>*p*-value = 0.887
**Mg**	*F* = 2.17<br>*p*-value = 0.122
**Mn**	*F* = 2.52<br>*p*-value = 0.085
**Ca**	*F* = 1.09<br>*p*-value = 0.398
**K**	*F* = 4.01<br>*p*-value < 0.05
**P**	*F* = 3.72<br>*p*-value < 0.05
**Na**	*F* = 7.72<br>*p*-value < 0.05
